# Costal cartilage as a promising technique for large bronchial fistula closure in fistulous empyema: a case report

**DOI:** 10.1186/s44215-024-00141-x

**Published:** 2024-02-26

**Authors:** Yoshiki Kato, Yasoo Sugiura, Hiroyuki Fujimoto, Toshinori Hashizume

**Affiliations:** 1 Respiratory Medicine, National Defense Medical College Hospital, 3-2 Namiki, Tokorozawa, Saitama, 359-8513 Japan; 2 Department of General Thoracic Surgery, National Hospital Organization, Kanagawa Hospital, 666-1 Ochiai, Hadano, Kanagawa 257-8585 Japan

**Keywords:** Bronchial fistula, Empyema, Costal cartilage, Endobronchial occlusion

## Abstract

**Background:**

There is no high level of evidence for the treatment of fistulous empyema. We report here a promising technique for closure of a bronchopleural fistula using costal cartilage as a bronchial embolus.

**Case presentation:**

The patient is a 79-year-old man. A computed tomography scan diagnosed a fistulous empyema associated with perforation of lung abscess of right middle lobe. After thoracoscopic curettage of the right empyema cavity, right middle lung resection and intercostal muscle flap coverage over the bronchial stump were performed. Seven months after the surgery, a bronchopleural fistula developed. Bronchoscopy revealed fistulas in the middle lobe bronchus and B3b. After the open window thoracostomy, the empyema cavity was cleaned up. The empyema cavity remained from the anterior to the lateral thoracic region of the second to fourth ribs. A part of the second through fourth ribs that formed the ceiling of the pleural empyema cavity was removed to create space for skin and thickened parietal pleura to fill the cavity. The costal cartilage obtained from the rib resection was trimmed and harvested to fit into the diameter of the bronchopleural fistula. The free costal cartilage was sutured and fixed with five stitches with 3-0 PDS. The visceral pleura was covered with the thickened parietal pleura and skin and fixed airtight. To maintain a tight seal, a Blake® silicone drain was inserted between the visceral pleura and the thickened parietal pleura, and a suction reservoir was utilized to sustain negative pressure. The drain was removed on the 21st day. As of 21 months postoperatively, the skin and thickened parietal pleura flap has maintained its integrity, and there has been no evidence of pus or recurrence of air leaks.

**Conclusion:**

In the case of a fistulous empyema extending anteriorly, costal cartilage can be easily harvested, making it a promising option as a lid for fistula closure.

## Background

There is no high level of evidence for the treatment of fistulous pleural empyema. The guidelines which have been published by the American Association for Thoracic Surgery describe the use of direct suture, soft tissue and omentum to close the fistula, but the evidence level is C [1]. Bronchial embolization is not included in the recommended treatment. We report here a new technique for closing a bronchial fistula using costal cartilage as a bronchial embolus after right middle lobectomy for lung abscess which caused fistulous empyema.

## Case presentation

**Fig. 1:**
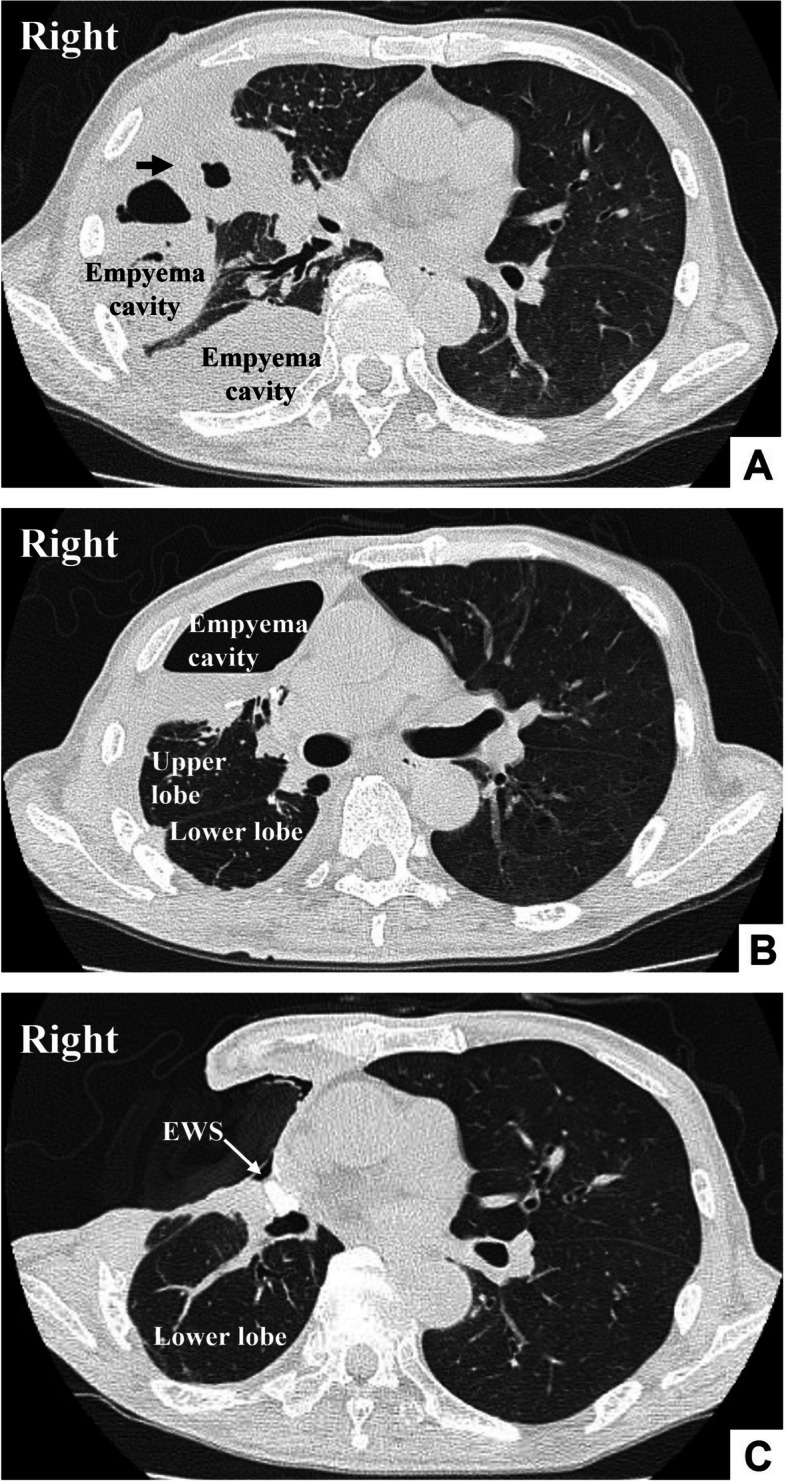
The computed tomography scan revealed a diagnosis of fistulous empyema, linked to the perforation of the right middle lobe abscess (arrow) (**A**). Following the thoracoscopic curettage of the right empyema cavity, we conducted a right middle lobectomy, and utilized intercostal muscle flap coverage over the bronchial stump was performed. Seven months after the surgical procedure, a bronchial fistula developed (**B**). After performing window open thoracotomy, our attempt at closing the bronchial fistula using multiple endobronchial Watanabe spigot (EWS) proved unsuccessful (**C**)

The patient is a 79-year-old man. Computed tomography scan revealed a diagnosis of fistulous empyema associated with perforation of right middle lobe abscess (Fig. 1A). After thoracoscopic curettage of the right empyema cavity, right middle lobectomy and intercostal muscle flap coverage over the bronchial stump were performed. Seven months after the surgery, a bronchial fistula developed (Fig. 1B). Bronchoscopy revealed fistulas in the middle lobe bronchial stump and B3b. After window open thoracotomy, an unsuccessful attempt was made to close the fistula using with endobronchial Watanabe spigot (EWS), but the EWS was dropped out of the body or was sputtered out through the mouth on coughing. Therefore, a combination of thoracoplasty and bronchial fistula plugging with costal cartilage was performed to close the fistula (Fig. 1C).

**Fig. 2:**
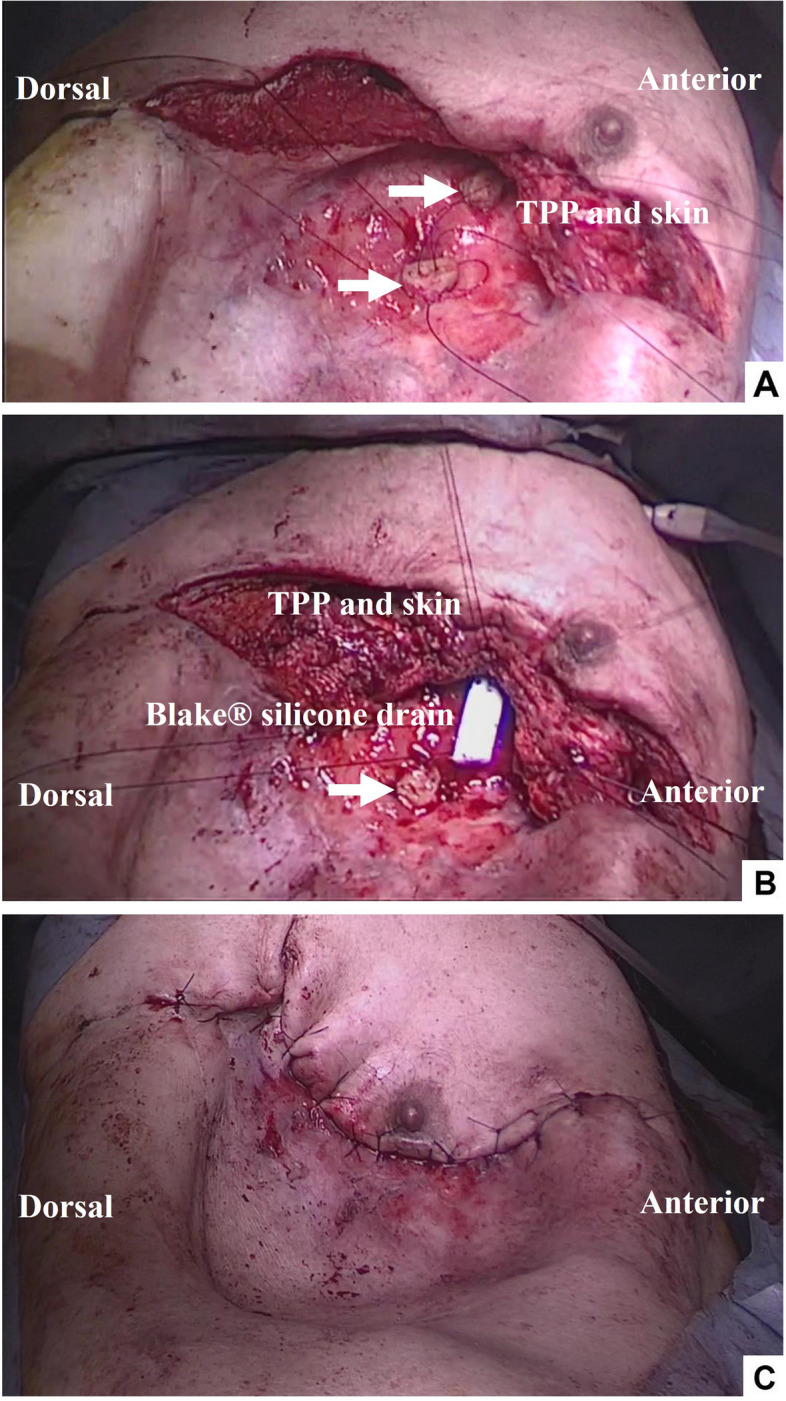
A part of the second through fourth ribs that formed the ceiling of the pleural empyema cavity was removed to create space for the skin and thickened parietal pleura (TPP) to fill the cavity. The costal cartilage obtained from the rib resection was trimmed and harvested to fit into the diameter of the bronchopleural fistula. The free costal cartilage (arrow) was sutured and fixed with five stitches with 3-0 PDS (**A**). A sealing test verified the absence of air leaks. The TPP and skin were used to tightly cover the visceral pleura, with 3-0 PDS fixing it airtight. To maintain a tight seal, a Blake® silicone drain was inserted between the visceral pleura and the thickened parietal pleura, and a suction reservoir was utilized to sustain negative pressure (**B** and **C**)

**Fig. 3:**
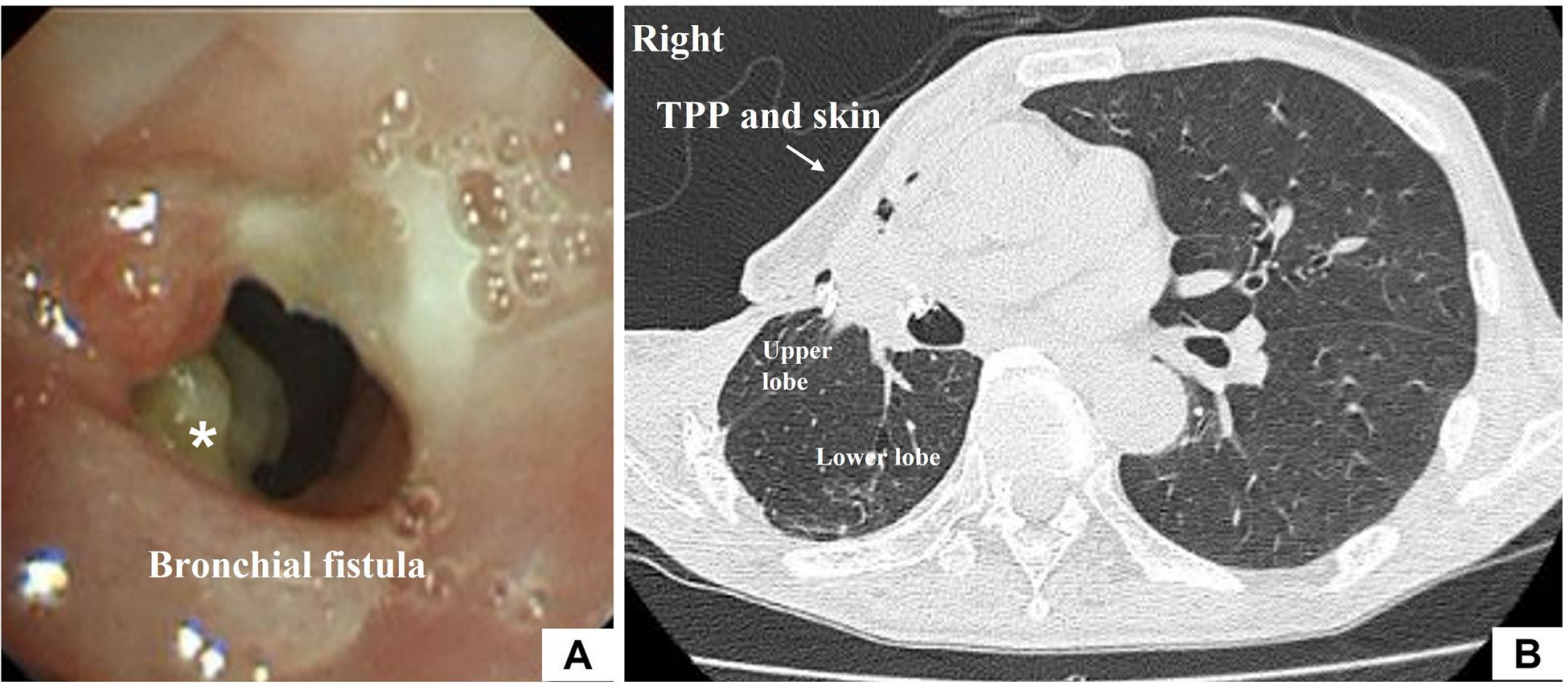
One month postoperatively, bronchoscopy revealed that the free costal cartilage was displaced (asterisk) (**A**). However, the covered thickened parietal pleura (TPP) and skin did not float, and there was no pus nor air leakage (**B**)

The bronchopleural fistula in the middle lobe was 2.5 cm in diameter, and the fistula in B3b was 1 cm in diameter. The empyema cavity remained from the anterior to the lateral thoracic region of the second to fourth ribs. A part of the second through fourth ribs that formed the ceiling of the pleural empyema cavity was removed to create space for the skin and thickened parietal pleura to fill the cavity. The costal cartilage obtained from the rib resection was trimmed and harvested to fit the diameter of the bronchopleural fistula. The free costal cartilage was sutured and fixed with five stitches with 3-0 PDS (Fig. 2A). A sealing test confirmed the absence of air leaks. The TPP and skin were used to tightly cover the visceral pleura, with 3-0 PDS fixing it airtight. To maintain a tight seal, a Blake® silicone drain was inserted between the visceral pleura and the thickened parietal pleura, and a suction reservoir was utilized to sustain negative pressure (Fig. 2B and C). In addition, the wound was compression fixed so as not to impede blood flow. On the seventh postoperative day, the compression fixation was removed. The drain was removed on the 21st day. One month postoperatively, bronchoscopy showed displaced rib cartilage (Fig. 3A), but the covered skin was firmly attached, and there was no pus nor air leakage (Fig. 3B). As of 21 months postoperatively, the skin and thickened parietal pleura flap has maintained its integrity, and there has been no evidence of pus or recurrence of air leaks.

## Discussion and conclusions

**Fig. 4:**
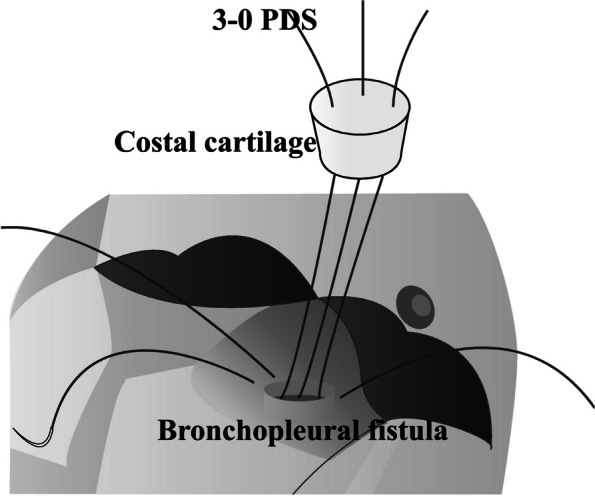
The advantage of rib cartilage is that it is easy to trim and thread the needle. The rib cartilage was trimmed to a size slightly larger than the fistula’s diameter. The needle penetrated the rib cartilage and sutured to through the bronchial wall and thickened pleura of the fistula. The fistula was then filled with rib cartilage and sealed with 3-0 PDS

The objective of this case report is to demonstrate the use of rib cartilage to close and successfully heal a fistulous empyema. The novelty and advantage of using rib cartilage is that large rib cartilage can be harvested easily, especially when there is an abscess cavity anteriorly. If the empyema cavity is located dorsally, this method is applied because the rib cartilage can be harvested if the wound is added over the costal cartilage. Furthermore, the advantage of rib cartilage is that it is easy to trim and thread the needle. Fig. 4 shows the technique for rib cartilage filling of the fistula. The rib cartilage was trimmed to a size slightly larger than the fistula’s diameter. The needle penetrated the rib cartilage and sutured to through the bronchial wall and thickened pleura of the fistula. The fistula was then filled with rib cartilage and sealed with 3-0 PDS. However, in general, cases that should be considered with caution, such as MRSA or nontuberculous mycobacterial mycobacteriosis, may be refractory to treatment [2].

In cases of empyema, the presence of a fistula is an independent prognostic factor and is notoriously intractable to treat [3]. The new technique presented here, a combination of closing a tracheobronchial fistula with rib cartilage and thoracoplasty, is based on the following two rationales. First, silicone spigot (EWS) developed by Watanabe et al. are often used as bronchial embolization devices [4]. In fistulous empyema, if the fistula can be closed using a bronchial embolus, open window thoracostomy can be avoided, or the thoracostomy wound can be closed [5–7]. Second, negative pressure drainage of the wound can remove exudate and edema, promote granulation and angiogenesis, control infection, and promote wound closure [8, 9]. Specifically, the fistula was closed using costal cartilage, which is autologous tissue and easily trimmed. Bronchial embolization stopped the air leak, a drain maintained negative pressure, and kept the thickened parietal pleura and visceral pleura tight and adhered together. Although bronchoscopy performed a months after the surgery showed the embolized costal cartilage was displaced, no recurrence of pus or air leak was observed even 21 months after the surgery because the thickened parietal pleura was in tight contact with the visceral pleura.

In a previous report, this technique was successfully used to close a 3.0-cm right main bronchial fistula after right pneumonectomy [10]. In another report, a 2.5-cm bronchopleural fistula that developed after right lower lobectomy was successfully closed by suturing the fistula with a rib attached to the intercostal muscles [11]. Suzuki et al. reported that they closed the fistula using a gastric seromuscular layer and omental pedicle flap [12]. The use of two soft tissues is useful in that it maintains airtightness and can be applied to close fistulas with large diameters. In addition, the use of an omentum may be advantageous in infected wounds. On the other hand, it is different from the present case. Methods of Suzuki et al. also require abdominal surgery. It may also be inappropriate for patients who have undergone gastric surgery or have already used omentum, and it may not provide sufficient omentum for patients who are significantly underweight. The method in the present case report can overcome these drawbacks.

In conclusion, in the case of a fistulous empyema with a residual cavity extending anteriorly, costal cartilage can be easily harvested, making it a promising option as a lid for fistula closure. Costal cartilage is advantageous in that it is easy to trim, easy to thread with fixation threads, easy to harvest multiple pieces of costal cartilage for multiple fistulas, and is autologous tissue.

## Data Availability

Data will be made available on reasonable request.

## Abbreviations

EWS: Endobronchial Watanabe spigot
TPP: Thickened parietal pleura
